# Adjunctive Telehealth Mindfulness Therapy for Persons With Dementia and Their Caregivers in the Rural Deep South

**DOI:** 10.1093/geroni/igab046.2179

**Published:** 2021-12-17

**Authors:** Lindsey Jacobs, Daniel Durkin, Michelle Hilgeman

**Affiliations:** 1 The University of Alabama, The University of Alabama, Alabama, United States; 2 The University of Mississippi, University, Mississippi, United States; 3 Tuscaloosa VA Medical Center, Tuscaloosa, Alabama, United States

## Abstract

The emotional care needs of persons with dementia (PwD) and their caregivers are multitudinous. Multicomponent interventions may be necessary to meet their multiple needs. Mindfulness interventions have a positive impact on well-being but are often only offered as a stand-alone treatment and typically are available only to the caregiver. This presentation will describe a telephone-delivered adjunctive mindfulness intervention that was offered to caregivers and dyads in conjunction with care consultation. Participants were 26 caregivers and 22 PwD living in the Deep South. The adjunctive mindfulness therapy included four core sessions and an additional five sessions that were optional. Mindfulness was deemed to be a “good fit” for almost 75% of the sample. Duration of mindfulness sessions ranged from 30 to 65 minutes. Participants attended more sessions as a dyad (M=10.10) compared to caregivers alone (M=6.5). Information regarding attendance and treatment engagement will be presented.

